# The nitrogen responsive transcriptome in potato (*Solanum tuberosum* L.) reveals significant gene regulatory motifs

**DOI:** 10.1038/srep26090

**Published:** 2016-05-19

**Authors:** José Héctor Gálvez, Helen H. Tai, Martin Lagüe, Bernie J. Zebarth, Martina V. Strömvik

**Affiliations:** 1 Department of Plant Science, McGill University, 21111 Lakeshore Rd, Ste-Anne-de-Bellevue, QC, H9W5B8 Canada; 2 Agriculture and Agri-Food Canada, Fredericton Research and Development Centre, PO Box 20280, 850 Lincoln Rd., Fredericton, NB, E3B 4Z7 Canada

## Abstract

Nitrogen (N) is the most important nutrient for the growth of potato (*Solanum tuberosum* L.). Foliar gene expression in potato plants with and without N supplementation at 180 kg N ha^−1^ was compared at mid-season. Genes with consistent differences in foliar expression due to N supplementation over three cultivars and two developmental time points were examined. In total, thirty genes were found to be over-expressed and nine genes were found to be under-expressed with supplemented N. Functional relationships between over-expressed genes were found. The main metabolic pathway represented among differentially expressed genes was amino acid metabolism. The 1000 bp upstream flanking regions of the differentially expressed genes were analysed and nine overrepresented motifs were found using three motif discovery algorithms (Seeder, Weeder and MEME). These results point to coordinated gene regulation at the transcriptional level controlling steady state potato responses to N sufficiency.

Potatoes (*Solanum tuberosum* L.), constituting the third most grown crop worldwide, usually have a sparse and shallow root system, and are therefore particularly sensitive to abiotic factors such as water and nutrient availability^1^. The macronutrient nitrogen (N) positively impacts potato biomass, tuber yield and quality, especially in fields with a limited natural supply^2^,^3^. However, excessive application of N can have two main undesirable effects: 1) decreased quality of the tubers which can render them less suitable for industrial food production^4^ and 2) leaching of nitrate into water supply systems and the emission of nitrous oxide, both of which can cause environmental damage^3^. Therefore, long-standing goals within the potato production sector are to increase plant N use efficiency as well as develop sustainable N management systems to optimize N supplementation to the amount required to maintain plant growth and achieve target yields^1^,^2^.

Whole transcriptome analyses using RNA-seq to examine genes involved in N deficiency responses have been done in maize^5^, *Arabidopsis*^6^, cucumber^7^ and rice^8^. Both a well-annotated reference genome and a reference transcriptome (gene models) are needed to carry out differential gene expression analysis using RNA-seq. The potato reference genome and transcriptome were initially published in 2011^9^,^10^. In 2012, a new annotation system (ITAG1.0) with updated gene models generated using new data from the tomato reference genome, was made available for both potato and tomato^11^,^12^. These resources have fuelled a renewed effort to analyse the molecular response of potato under different biotic and abiotic conditions^13^.

N related regulatory motifs have been identified in maize and *Arabidopsis thaliana* genes^14^,^15^ and point to coordinated responses to nitrate at the transcriptional level in plants. One of these motifs, the Nitrate Related *cis-*Element (NRE) identified in *Arabidopsis*, has also been found in the promoter region of the Nitrite Reductase (*NIR*) gene of several monocotyledonous and dicotyledonous plants such as spinach, tobacco, rice, maize and sorghum^15^. However, few studies have been done on regulatory motifs in the upstream regions of genes in potato. A study analysing the level of expression of a transgenic patatin class-I and β-glucuronidase (GUS) chimeric gene in field-grown potato found significant changes in expression depending on the promoter used in the construct and the regulatory motifs it contained^16^. These results highlight the need to further study and understand potential gene regulation mechanisms in potato, especially in response to critical abiotic factors such as N sufficiency.

Additionally, transcriptome analysis can be used for the discovery and annotation of overrepresented motifs. *De novo* motif discovery algorithms such as Seeder^17^ have been used before to predict the binding sites of regulatory elements in the upstream flanking regions of genes in other plant species^18^,^19^. Differences in the 5′-upstream flanking regions of potato genes, including variations in the number and types of regulatory motifs, have also been correlated with changes in gene expression^16^.

Transcriptome analysis can also be applied as an alternative method for quantifying N sufficiency^20^,^21^,^22^. Expression profiles associated with N sufficiency can be used to guide decisions on N fertilizer application in potato fields. Other technologies proposed for nutrient monitoring in crops include biosentinel plants that use promoters from nutrient responsive genes to drive reporter genes^23^. Both of these approaches can be enhanced through transcriptome analysis.

The current study uses RNA-seq data generated from three commercial potato cultivars (Shepody, Russet Burbank and Atlantic) to examine the steady state transcriptome response of potato to N supplementation. Genes with expression that was affected by the rate of supplemented N were further analysed for overrepresented DNA motifs in their upstream flanking regions through *de novo* motif discovery analysis. In all, 39 genes were differentially expressed in all three cultivars, and in total, nine potential nitrogen responsive motifs were identified.

## Results

### Effects of N supplementation on potato plants

The availability of N in the soil is known to cause measurable changes in certain characteristics of potato plants including dry biomass at harvest, fresh tuber yield, and chlorophyll content^3^. To determine the effects of N sufficiency, two contrasting rates of N supplementation were applied (0 kg N ha^−1^ and the recommended rate of 180 kg N ha^−1^) in a randomized complete block design (Table 1). Four replicated blocks were used. All trait measurements were statistically tested with a two-factor Analysis of Variance to determine the significance of the observed changes among plants from different cultivars grown under different N supplementation rates (Table 2, Supplementary Tables 1 and 2).

**Figure 1:**
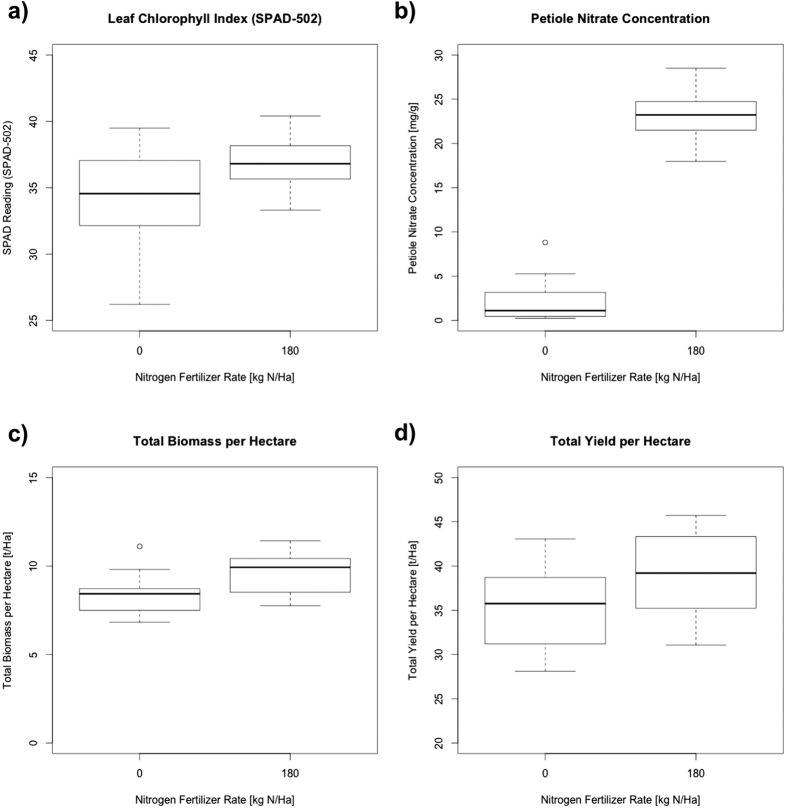
Comparison of four phenotypic traits in potato plants grown at two different N supplementation rates. Plots are showing different phenotypic measurements in potato plants from three cultivars (Shepody, Russet Burbank and Atlantic) grown at two rates of N supplementation (0 kg N ha^−1^ and 180 kg N ha^−1^). In all cases, plants with no N supplementation display signs of N deficiency and early senescence. (**a**) Relative leaf chlorophyll content measured by light transmittance using a SPAD-502 meter. (**b**) Petiole nitrate concentration measured colorimetrically. (**c**) Total plant biomass, measured from plant components (tubers, vines, stolons plus readily recoverable roots) for a representative sample of plants. (**d**) Total fresh tuber yield in the field from a representative sample of plants.

The chlorophyll content index was measured in foliar tissue samples collected from the field grown plants using Special Products Analysis Division (SPAD) readings. Plants without N supplementation had significantly lower SPAD readings than those grown with supplemented N (Fig. 1a). This result indicates that plants grown without added N had lower concentrations of chlorophyll in their foliar tissue, which is indicative of reduced N sufficiency. The SPAD readings among plants of different cultivars were also significantly different.

Petioles were collected from the same leaves used for the SPAD readings and the concentration of petiole nitrate was chemically determined for each biological replicate. Petioles collected from plants without supplemented N had significantly lower concentrations of petiole nitrate than did the plants grown with the addition of N (Fig. 1b). There were no significant differences in the petiole nitrate concentrations among plants of different cultivars.

The effects of N on biomass were measured before vine desiccation. To calculate the effect of N supplementation on biomass, whole plants were sampled and partitioned into vines, tubers, stolons and readily recoverable roots. Using information on the spatial distribution of plants in the field as well as the dry matter weight of the sampled plants, total biomass per hectare was calculated (Fig. 1c). The results show that biomass significantly increased in the groups grown with supplemented N. Dry matter weight also differed significantly among the three cultivars. However, the differences in biomass between cultivars were less pronounced than those observed due to N supplementation.

Finally, fresh tuber yield measured at harvest was analysed to determine the effect of N supplementation on yield. Tubers from the plants collected at harvest for biomass determination were also washed and weighed before drying. The total fresh yield for tubers was calculated using additional information on the spatial distribution of plants in the field (Fig. 1d). Tuber yield varied significantly among the three cultivars. N supplementation also had an observable effect on fresh tuber yield, although not as pronounced as the cultivar effect.

### Transcriptome sequencing reveals N responsive genes

Sequencing of RNA samples was carried out to determine the differences in the transcriptomes of plants grown under two N treatments, deficient and sufficient, to obtain a list of N responsive differentially expressed genes. Total RNA was extracted from the foliar tissue samples collected from the same apical leaflet used for SPAD readings and petiole sampling. Paired-end sequencing (2 × 100 cycles) of the prepared RNA libraries was performed using a HiSeq 2000 [Illumina]. Sequencing results were trimmed and filtered for quality, and aligned to the potato reference genome^10^ using TopHat^24^.

The transcriptomes of plants grown with and without supplemented N at 180 kg N ha^−1^ were compared to find differences in gene expression that were highly specific to N status and that were consistent across cultivars and developmental time points. Samples were collected from three cultivars (Shepody, Russet Burbank and Atlantic) at two developmental time points: eight and ten weeks after planting. Sampling of the four replicated blocks was done over the course of a day (i.e. 0800 h, 1100 h, 1400 h and 1700 h for blocks 1 to 4 respectively) to allow for the removal of genes with significant time of day variation in expression in the analysis.

Aligned reads were analysed using CuffDiff^25^ and lists of differentially expressed genes in plants grown under different N treatments were made for each cultivar and developmental time point. Gene lists were compared to find genes with similar expression patterns across all cultivars and developmental time points. The experimental design was focused on identifying genes involved in steady state responses to N supplementation. Differentially expressed genes that were common among the cultivars and development time points were divided into two groups: those over-expressed in plants grown with supplemented N and those under-expressed in plants with supplemented N. A summary of the number of genes found to be differentially expressed in each analysis can be found in Supplementary Table 3.

In all, 30 genes were consistently over-expressed (Table 3) and nine genes under-expressed (Table 4) with N supplementation, across cultivars and over developmental time points. Differences were found in gene expression among cultivars and developmental time points, but they were not the focus of the current study. For example, 51 genes were over-expressed in only one of the two time-points and another four genes were under-expressed in only one of the two time-points (Supplementary Table 4). Alternative splicing analysis was also performed on the raw RNA-seq data, but did not reveal alternate splicing events in the 39 genes found to be N responsive in all cultivars (data not shown).

**Figure 2:**
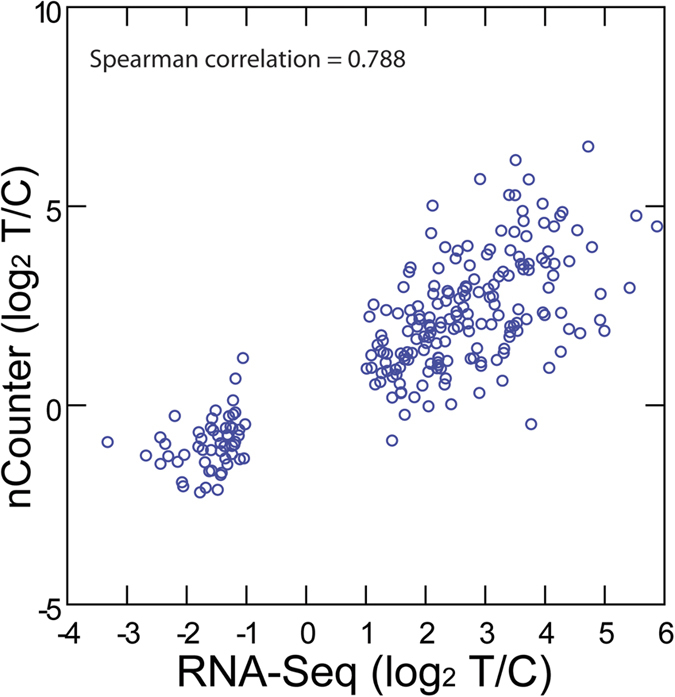
Correlation between gene expression measured using RNA-Seq and an nCounter Digital Analyzer for 39 differentially expressed genes. Spearman rank correlation (Morey *et al.* 2006) of the log_2_ differences between RNA-seq reads (FPKM) and nCounter Digital Analyzer measurements for the 39 genes that were found to be differentially expressed in three potato cultivars (Shepody, Russet Burbank and Atlantic). Two groups are formed: the group in the bottom left represents the measurements that correspond to the 9 genes found to be under-expressed in plants with N supplementation; the group in the top right corresponds to the 30 genes found to be over-expressed in plants with N supplementation.

To validate the differential expression of the shared genes, the RNA samples were tested again using an nCounter Digital Analyzer (Nanostring Technologies, Inc.). Probes were designed for the 39 differentially expressed genes previously identified through RNA-seq. The nCounter reads were normalized using the expression of five housekeeping genes as a reference^26^,^27^. A Spearman Rank correlation was used to compare the measurements obtained from the nCounter Digital Analyzer with previous data generated through RNA-seq (Fig. 2). A positive correlation (r = 0.79) was found between the methods.

The N responsive genes were annotated using Gene Ontology (GO) terms and KEGG pathway information to determine if they have known functions in common. The GO information was obtained from the collected results of five GO term analysis pipelines (Trinotate (HMM and BLAST), OrthoMCL-UniProt, BLAST2GO, Phytozome and InterPro2GO) on ITAG1.0 genes^28^. GO terms were found for all but six of the N responsive genes. The KEGG pathway information and Enzyme Commission (E.C.) numbers of the genes were obtained from the SolCyc database in the Sol Genomics Network^12^. Out of the ten genes with associated E.C. numbers, seven were part of well-defined metabolic pathways (Tables 3 and 4): *Alanine-glyoxylate aminotransferase* (Sotub10g018540), *Glutamate decarboxylase* (Sotub01g005580), *Glutathione S-transferase* (Sotub10g024560), *Sulfate adenylyltransferase* (Sotub09g024290), *Proline dehydrogenase* (Sotub02g033320), *Primary amine oxidase* (Sotub08g025870), and *Ubiquinone/menaquinone biosynthesis methyltransferase* (Sotub12g020880).

Most of the KEGG pathways associated with the differentially expressed genes are involved in amino acid metabolism. In addition, one gene is directly associated with sulphate reduction pathways (*Sulfate adenylyltransferase* Sotub09g024290), as well as three other sulphate-related genes (*Sulfate transporter* Sotub04g021910; *High affinity sulfate transporter 2* Sotub04g027100; *High affinity sulfate transporter 2* Sotub10g013960) that have been previously reported to have an N response in *Arabidopsis* and tobacco^29^,^30^. The Gene Ontology analysis revealed a very wide variety of GO-terms associated with the differentially expressed genes. The most commonly found GO term was related to an integral component of the cell membrane (GO: 0016021). Other GO terms associated with the 39 differentially expressed genes included response to cadmium ion (GO:0046686), oxidation-reduction process (GO:0055114) and transmembrane transport (GO:0055085).

### Overrepresented sequence motifs are present in the upstream flanking regions of nitrogen responsive genes

To predict potential N responsive regulatory mechanisms in the 39 differentially expressed genes, the 1000 bp upstream flanking regions were analysed for putative regulatory DNA motifs. *De novo* motif discovery tools Seeder^17^, Weeder^31^ and MEME^32^ were used to identify overrepresented motifs in the upstream flanking region of the differentially expressed genes. Each program works by implementing a completely different motif prediction algorithm, and analysing the sequences using all three tools increases the probability of finding more overrepresented motifs.

To discover overrepresented motifs, both Weeder and Seeder require a background file that contains information on k-mer frequencies in the relevant region or regions of the genome. Because motif discovery was focused on the upstream flanking region of N responsive genes, the reference background was computed from the sequences of the upstream flanking regions of all the genes in the Potato Reference Genome^10^. These sequences were obtained using the Transcription Start Site (TSS) and strand information for all the genes found in the ITAG1.0 annotation system^11^,^12^.

Seeder and MEME assume that overrepresented motifs will appear in all or most of the input sequences. This means that if one or several sequences do not contain an overrepresented motif, it might not be discovered. By carrying out multiple runs of motif discovery separately in smaller random subsets, there is an increased probability of finding motifs that are not present in all the sequences of the original list. Therefore, the two lists of upstream flanking regions of N responsive genes were sub-divided into smaller random sub-groups of 10 sequences and these smaller subsets were used as input for MEME and Seeder. Due to the nature of its algorithm, it is not necessary to use random sub-groups with Weeder and therefore a single list of upstream flanking regions was used as input for this program.

It is common for motif discovery programs to predict motifs that are very similar to each other, making it difficult to distinguish redundant and unique hits. This problem is further complicated when using random sub-groups as input, because the same motif might be present in several different sub-groups and will therefore appear multiple times in the final output. To overcome this issue, predicted motifs were clustered using the k-medoids clustering algorithm found in the TAMO package^33^ and the average of every cluster was then used as a representation of that cluster. The final motifs discovered in both lists of differentially expressed genes, after clustering, are summarized in Table 5.

Two databases of experimentally validated DNA-motifs, JASPAR^34^ and PLACE^35^, were consulted to determine whether any of the predicted motifs had previously reported functions that might have any relationship with N response. All discovered motifs returned at least one significant result from each of these databases, however none of those results matched the computationally discovered motif identically. Most of the results found in the databases differed with the discovered motifs by one nucleotide in the aligned region.

The results obtained from searching for motifs in the PLACE database can be used to predict regulatory mechanisms in potato because this database aggregates only experimentally validated motifs in plants. The matches from PLACE had several associated biological functions including the regulation of histone, auxin, and amylase genes (Table 5). A regulatory motif similar to one discovered by Weeder (Motif 3) has been previously shown to regulate patatin production in potato tuber, possibly through modulation with exogenous sucrose^36^. Also, three motifs discovered by Seeder (Motifs 5, 6, and 9) matched with PLACE motifs that have been associated with light-responsive promoters: 3AF1^37^, S1F^38^ and GAPB^39^.

Because the NRE motif was described in previous studies^15^ but was not found with our motif discovery process, an attempt was made to find instances of this motif in the upstream regions of our 39 N responsive genes. None were found. The NRE motif was identified in the promoters of *NIR* genes in plants, however no *NIR* genes were identified as N responsive in the current study, because developmental stage and time of day variable genes were removed. The upstream flanking regions of three potato *NIR* genes (Sotub01g045870, Sotub08g028180, and Sotub10g014930) were therefore inspected and the NRE motif was indeed found in two of those regions. This indicates that the NRE motif is conserved in the upstream regions of the *NIR* genes in potato and therefore possibly involved in N response specific to developmental stage or time of day. However, there is no evidence in the current study to suggest that it regulates the steady state N responsive genes identified here.

**Figure 3:**
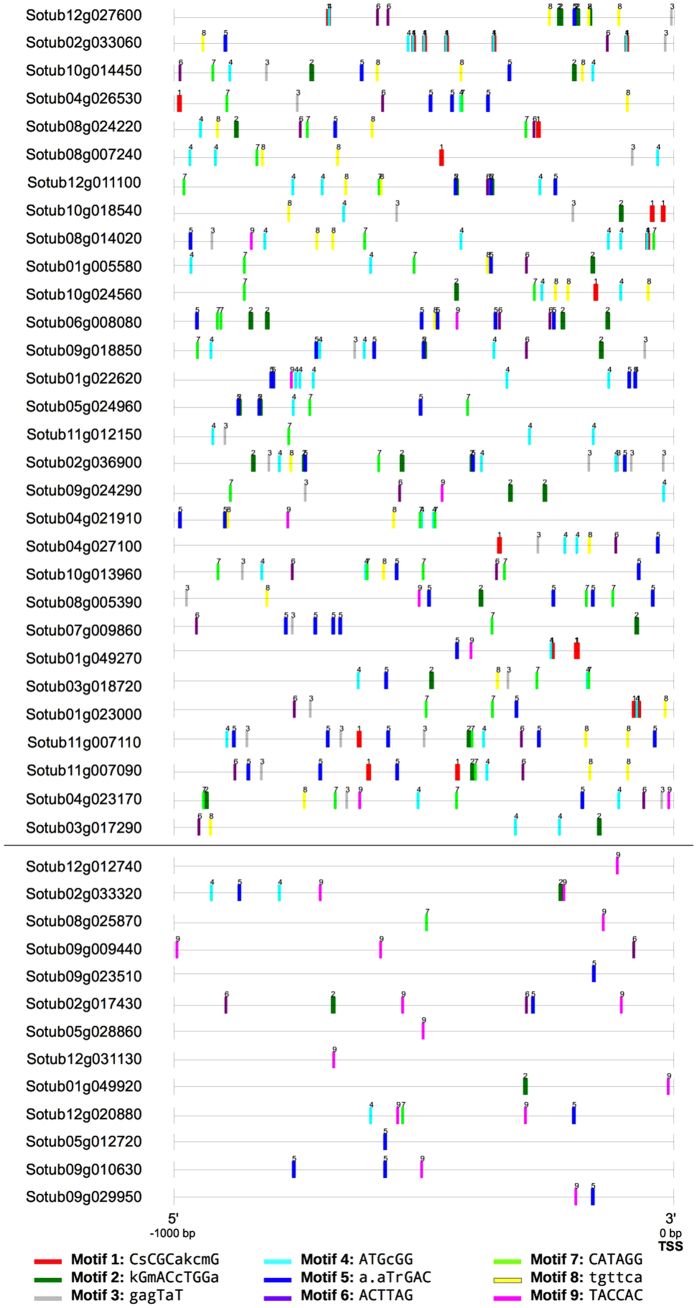
Motif locations in the 5′-upstream flanking region of N responsive genes. Diagrams representing the 1000 bp 5′-upstream region of the 30 over-expressed (top section) and 13 under-expressed (bottom section) N responsive genes. Coloured rectangles indicate an instance of a discovered motif in that position; one-letter representations of every motif are found in the diagram key at the bottom. The Transcription Start Site (TSS) of every gene is located at the right end of each upstream flanking region.

Every discovered motif was mapped back to all upstream flanking regions in the N responsive genes. This served two purposes: it enabled the identification of additional redundant motifs, which mostly mapped to the same positions within the flanking regions, and it also permitted the comparison of upstream flanking regions from genes with similar reported functions. Figure 3 contains diagrams showing the position of all motif instances in the 5′-upstream flanking regions of all N responsive genes. In general, the motifs that appear the most times in the upstream-flanking regions of all N responsive genes are Motif 4 [ATGCGG] (62 times), Motif 5 [A.ATrGAC] (56 times) and Motif 7 [CATAGG] (40 times).

## Discussion

The effect of N on the growth and yield of potato has been the subject of several studies, including those focused on the phenotypical effects of N-deficiency^40^,^41^ and more recently, those that have analysed the effects of N on the expression of a small subset of genes^20^,^21^,^26^. However, a modern tool like RNA-seq offers a new way to compare the whole transcriptome of potato plants grown under differing N status. Our experiment is one of the first to use RNA-seq to find differentially expressed genes in plants grown in the field, with and without N supplementation.

The current study found that the three potato cultivars examined responded in the same way to supplemented N. The results were increased tuber yield, significantly greater N uptake and dry biomass, as well as greater leaf chlorophyll content. Gene expression analysis identified genes responding to N sufficiency similarly across three cultivars and two developmental time points. Previous studies have shown that genes can be variably responsive to N depending on the time of day^42^. The experimental design of the current study removes genes with time of day variation, and leaves only genes that consistently show differential expression. The final set of 39 identified genes were those that showed a consistent response to N supplementation in all three cultivars throughout the day, both at eight and ten weeks after planting.

Our previous studies examined expression of potato genes involved in N uptake, assimilation and transport, and demonstrated that an ammonium transporter gene, *AMT1*, was responsive to N rates over different developmental time points^21^. However, this gene was found to have variation in expression at different times of the day^42^ and therefore did not meet criteria for screening in the current study. Functional analysis of the differentially expressed genes in the current study indicated association with KEGG pathways involved in amino acid metabolism. Two over-expressed genes included the aminotransaminases *Aminotransferase-like protein* (Sotub12g011100) and *Alanine-glyoxylate aminotransferase* (Sotub10g018540). Additionally, *Proline dehydrogenase* (Sotub02g033320) was found to be under-expressed with N supplementation. Decreased proline dehydrogenase activity was also found under conditions of N supply in French bean^43^, which concurs with the results of this study. The action of proline dehydrogenase and pyrroline-5-carboxylate dehydrogenase lead to degradation of proline to glutamate.

There is also evidence for regulation of C:N balance with N supplementation. Over-expressed genes encoded enzymes functioning in both C and N metabolic pathways including two enzymes in the GABA shunt: *Aminotransferase-like protein* (Sotub12g01110) and *Glutamate decarboxylase* (Sotub01g005580). The GABA shunt is involved in the regulation of C:N balance in plants. GABA may also have roles related to stress response and as a signalling molecule^44^. Another gene involved in both C and N metabolism was *Alanine-glyoxylate aminotransferase* (Sotub10g018540), which converts alanine and glyoxylate to glycine and pyruvate. This enzyme is involved in photorespiration in *Arabidopsis*^45^ and high levels of photorespiration are associated with low alanine and high glycine^46^. Interestingly, photorespiration is also linked to increased nitrate assimilation^47^.

Additionally, four sulfate-related genes were found to be N responsive, one of which was part of the sulfate reduction and sulfate activation pathways, indicating a potential relationship between the sulfate and nitrate metabolic pathways. This type of relationship has been observed before in tobacco where N availability has been shown to regulate ATP sulfurylase^29^ and, more recently, in *Arabidopsis* where sulfur transporter *SULTR1;1* is found to be down-regulated in conditions of N insufficiency^30^.

GO-terms associated with N responsive genes were found to be related to transmembrane transport. These genes were involved in transport of sulfate, nitrate, amino acids and peptides; which correspond with the differential expression observed in amino acid and sulfate metabolism genes. Under-expression of a cation transporter and over-expression of a cation transport regulator were also found. These results indicate that proton movement may be involved in responses to N supplementation. Cation transport can affect the activity of the GABA shunt enzyme Glutamate decarboxylase, which is controlled by pH and Ca^2+^-calmodulin^48^.

The Flowering locus T protein gene (Sotub05g028860) in potato plays a role in controlling maturity and tuberization^49^. This gene was under-expressed in plants with supplemented N, suggesting that increased N sufficiency can delay maturity. Two genes that were also under-expressed are similar to *Arabidopsis* genes involved in N response: a nodule inception protein similar to the *Arabidopsis* NIN-like transcription factor (Sotub01g049920) and Nodulin (Sotub05g012720). The former is a nitrate responsive transcription factor^50^ and the latter participates in nodule formation in legumes, and in amino acid transport in non-leguminous plants^51^.

The N responsive genes were shown to have shared motifs in the upstream promoter regions, which supports the idea of coordinated regulation at the transcriptional level of genes responding to N supplementation. Motif discovery was carried out based on a previously described strategy that sub-divides genes into random subgroups to increase the probability of finding overrepresented motifs that are not present in all flanking regions (Munusamy *et al.*, unpublished). Initial results of the motif discovery algorithms produced many redundant motifs, which was expected because different sub-groups may contain instances of the same motif. Therefore, a k-medoids clustering strategy was used to reduce the incidence of redundant motifs. In the end, the average motif of each cluster was taken as the representative motif and used in the subsequent annotation and mapping analyses.

Annotation of discovered motifs using experimentally validated motif databases, especially PLACE, revealed several intriguing putative regulatory mechanisms. Three of the overrepresented motifs in the upstream regions of N-responsive genes, including Motif 5 which was one of the motifs most frequently found in this dataset, were similar to motifs previously found to be associated with light-responsive genes^37^. Additionally, Motif 3 [GAGTAT/ATACTC] is very similar to a previously reported motif [AATACTAAT] involved in the regulation of patatin production in tubers as a response to exogenous sucrose^36^. These results are very similar to a previous study focusing on the regulation of patatin genes in potato^16^, which further suggests that C:N balance regulation is involved in the response to N supplementation.

The motif discovered by Seeder in the 5′-flanking region of under-expressed genes (Motif 9 TACCAC) is very similar to the binding site of transcription factor S1F [TACCAT], a *cis*-regulatory element associated in the down-regulation of plastid related genes such as *rbc*S, *cab*, and *rp1*21.The introduction of this binding site into transgenic tobacco plants has been experimentally shown to cause the differential repression of the *rps*1 plastid gene in non-photosynthetic tissue^38^. The similarity of the predicted motif and the experimentally validated one suggests that lower concentrations of available N could be potentially triggering a repression of plastids in the foliar tissue.

Mapping of predicted motifs in the upstream flanking regions of N responsive genes revealed few obvious patterns in the incidence of motifs with relation to the Transcription Start Site (TSS). Motifs seem to have no defined position within the upstream flank, and there are several instances of motifs appearing multiple times within the same region, which is not uncommon in other organisms. The upstream flanking regions of two over-expressed genes (Sotub11g007110 and Sotub11g007090) stand out due to their similarity, both in number and location of predicted motifs. Interestingly, both genes have the exact same annotation in the ITAG1.0 system (protein of unknown function) and are located close to each other on the same chromosome. The similarity of the positions of the discovered motifs in the upstream-flanking regions of these genes indicates a similar molecular mechanism regulates the expression of both, and also suggests that they share very similar, if not identical function. By analysing these genes and searching for a similar distribution of motifs in the upstream flanking region of at least one other gene, it might be possible to finally identify the function of these unknown proteins as well as predict their relationship with N supply in potato.

Upstream regulatory motifs in N responsive genes have previously been found in other species, such as maize^14^. These genes included nitrate, nitrite and ammonium transporters; nitrate and nitrite reductases; as well as glutamate and glutamine synthases. Nitrate transporter was the only gene in common between the maize study, which identified genes expressed at 4 h after N treatment, and the present study, which identified genes expressed at steady state nitrogen supplementation. Therefore, it is not surprising that the putative regulatory motifs found in our study are different than those in the study in maize.

There was no observed similarity between the motifs found in the upstream regions of the 39 N responsive genes in potato and the NRE motif found in the upstream region of the nitrite reductase gene *NIR1* of *Arabidopsis* and several other plants^15^. The *NIR* genes were not among the 39 genes that were differentially expressed in the current study. Previously, the authors have shown that a potato *NIR* gene had time of day^42^ and developmental stage^21^ variations and this is likely why it is not among the 39 genes. The current study focused on genes without time of day variation, and of longer term responses to N supplementation, with gene expression measurements at eight and ten weeks after planting. Additionally, because the experiment was done in the field, plants that did not receive N supplementation were not completely starved of N. The results demonstrate that longer term, steady state responses to N involve a different set of genes than shorter term, time of day variable responses, hence, regulatory motifs are also different. However, the presence of NRE in the upstream region of *NIR* genes in several different plant species (including potato) raises the possibility that the putative motifs found in this study could also be present in the N responsive genes of other plants.

In conclusion, our study provides evidence for regulatory coordination of steady state responses to N sufficiency at the level of gene expression in potato. These results have many potential applications including development of N status monitoring systems.

## Materials and Methods

### Plant Materials and Growth Conditions

Potato plants were propagated in the field at the Fredericton Research and Development Centre of Agriculture and Agri-Food Canada, Fredericton NB, Canada in 2012. The experiment included two fertilizer N rates (0 and 180 kg N ha^−1^) in a randomized complete block design with four blocks. Fertilizer N was banded at planting as ammonium nitrate (34-0-0). All plots also received 150 kg ha^−1^ of P_2_O_5_ and K_2_O banded at planting. Plots were six rows (5.46 m) by 8 m in size where the outer rows were guard rows.

The experiment was planted on May 23 using 0.91 m row spacing and 0.3 m within-row spacing. A modified planter was used to band the fertilizer treatments and open the rows. Hand-cut 50 g seed-pieces each of cultivar Atlantic (U.S. Department of Agriculture, 1978), Russet Burbank (L. Burbank, approx. 1880) and Shepody (Agriculture Canada-New Brunswick, 1969) were hand-planted and imidacloprid was applied to control for Colorado potato beetle. The seed-pieces were covered using discs. One hill of cv. Chieftain was planted at the end of each row to avoid edge effects.

Sampling was done on July 25 and August 8. At each sampling, the apical leaflet of the last fully expanded leaf (usually the fourth leaf from the top of the plant) was sampled from each of 15 randomly selected plants in each plot and placed into 50 ml Falcon tubes. The tube was immediately placed in liquid N, and stored at −80 °C until RNA extraction. Petioles were then collected from the same leaf for determination of petiole nitrate concentration.

### Leaf Chlorophyll Measurement and Petiole Nitrate Determination

Leaf chlorophyll index (LCI-S) was measured on the apical leaflet of the last fully expanded leaf, which was also used to measure gene expression, using a SPAD-502 reader (Konika Minolta). The LCI-S was determined in the section of the leaf midway from the mid-rib to the leaf margin^41^. Petioles were dried at 55 °C and ground to pass a 2 mm screen. A 0.2 g subsample of petiole tissue was extracted with 40 ml distilled water and a 15 min shaking time. The concentration of NO_3_-N in the extract was determined colorimetrically using a Quikchem 8500 flow injection analyzer (Lachet) using QuikChem method 90-107-04-2-A^41^. Two-factor ANOVA of petiole nitrate determination and SPAD readings between the groups was calculated using the R statistical language v. 3.1.1.

### Dry Biomass and Fresh Yield Determination

Whole plants were sampled before vine desiccation to determine their dry biomass content and at harvest to measure fresh tuber yield. Each plant was separated into vines, tubers and stolons as well as any recoverable roots. Tubers with a diameter below 0.5 cm were left as part of the stolons and roots. Vines, stolons and roots were washed, weighed and then oven-dried. After drying, they were weighed again and the dry matter of each sample was determined. Tubers were washed and weighed to determine fresh yield. Finally, tuber samples were taken from every experimental group and quartered along the long axis. One quarter of every tuber was randomly selected and sliced into 1 × 1 cm strips, and then weighed before and after oven-drying to determine dry matter content^52^. Two-factor ANOVA of dry biomass and fresh tuber yield between the groups was calculated using the R statistical language v. 3.1.1.

### RNA Extraction

Leaf tissue was ground to a powder in liquid N using a mortar and pestle. Samples were pre-extracted in 1 ml Hot Borate Buffer^26^. The lysate supernatant (200 μl) was used for RNA extraction with the Biomek NXP Laboratory Automation Workstation (Beckman Coulter) using the RNAdvance Tissue Kit (Agencourt) according to the manufacturer’s instructions for liquid samples. The RNA concentration and quality were determined using a NanoDrop 1000 Spectrophotometer (Thermo Scientific) and 2100 Bioanalyzer (Aglient Technologies) respectively.

### Library Preparation and Sequencing

Libraries were generated using the TruSeq RNA kit (Illumina). Messenger RNA was purified from 1 μg of total RNA using oligo-dT beads. The mRNA enriched fraction was reverse transcribed to generate cDNA fragments that were sheared to yield ~200 bp fragments. Following end-repair, 3′ end adenylation steps, and index ligation, a PCR amplification step was performed. A separate index was used for each N treatment-variety-replicate combination for a total of 24 indices.

Two lanes of sequencing were done for each 24 index multiplex, one for each time point. The quality of the library was assessed on a DNA 1000 chip and quantified by qPCR. Libraries were subjected to 100 bases of sequencing on a HiSeq 2000 (Illumina) instrument in paired-end mode. Initial quality control of the data was performed using the software included with the sequencer.

### Genome Alignment and Differential Expression Analysis

Output from the sequencer was aligned to the *S. tuberosum* reference genome v3_2.1.10^10^ using the TopHat software suite v. 2.0.9^25^ with the mode for “fr-unstranded” library types. The quality of the alignments was verified using the ‘flagstat’ tool from the SAMtools software suite v. 0.1.19^53^.

Reads were assembled into transcripts with CuffLinks v. 2.1.1^54^, using the *S. tuberosum* ITAG1.0 annotation file obtained from the *Sol Genomics Network*^11^,^12^. Transcriptome assembly was performed with the ‘multi read correct’ and ‘fragment bias correct’ modes activated. Finally, assembled transcripts from different replicates and treatments were merged into a single reference transcriptome for each variety using the ‘CuffMerge’ tool included in CuffLinks.

Differentially expressed genes were identified for each time-point and each cultivar using CuffDiff^55^. The same *S. tuberosum* reference genome as well as the single, merged transcriptome were used as reference for differential gene expression. Finally, genes found to be differentially expressed in each cultivar, were compared using custom perl scripts and a single list of over-expressed and under-expressed genes found in all cultivars at both time-points was produced.

### Expression Analysis using nCounter Digital Analyzer

The 39 genes with significant differences in expression from the CuffDiff analysis were selected and used to validate the gene expression results. The same RNA samples indicated above were prepared using the reagents and method described in Geiss *et al.* 2008 for the nCounter (Nanostring Technologies) multiplex gene expression analysis. The nCounter data was adjusted according to the manufacturer’s instructions using the manufacturer-provided spiked positive and negative controls. Gene expression of five housekeeping genes 18S rRNA, actin, cyclophilin^56^, elongation factor 1-α (EF-1-alpha)^57^ and cox1-B^20^ was also measured and the geometric mean of their expression was used to normalize gene expression values for the 39 test genes^26^,^27^. Spearman rank correlation was performed using SYSTAT v. 13 (Systat Software) and used to compare expression data from nCounter and transcriptome sequencing for the 39 genes.

### *De Novo* Motif Discovery

A FASTA file containing the 1000bp upstream flanking regions upstream of the transcriptional start sites for all the genes in the *S. tuberosum* reference genome v3_2.1.10^10^ was generated using the ‘faidx’ tool of the SAMtools software suite v. 0.1.19^53^. The transcription start-site and strand information for every gene were obtained from the ITAG1.0 annotation file^11^,^12^. From this general file, two subsets were created, each containing only the upstream flanking regions of the genes that were found to be significantly over-expressed or under-expressed in response to supplemented N. Motif discovery was performed separately for each set of differentially expressed genes.

Three different programs were used for *de novo* motif discovery: Seeder v. 0.01^17^, MEME v. 4.10.0^32^ and Weeder v. 2.0^31^. All three motif discovery programs were run simultaneously on *Guillimin*, Mcgill University’s high-performance computing server (http://www.hpc.mcgill.ca/), using high-memory computing nodes. A series of 5000 random subsets containing 10 random promoters each were generated for each differentially expressed gene list these random subsets were then used as input for the motif discovery programs Seeder and MEME.

The FASTA file containing all 1000 bp upstream flanking regions in the genome was used to generate the background files in Seeder and Weeder. All motif discovery programs were run to find motifs with a minimum length of 6 bp in both the forward and reverse-complement strands.

Significance of predicted motifs was determined differently for each algorithm, based on the available parameters reported by the program: in Seeder, a maximum q-value of 0.05 was allowed; in MEME, a maximum e-value of 0.001 was allowed; finally, only the top five results of each Weeder run were considered significant.

### Motif Annotation and Mapping

All significant motifs were converted into the same format for comparison and annotation using the TAMO software suite^33^. Redundant motifs were clustered together using k-medoids algorithm, as implemented in TAMO. Cluster averages were uploaded to STAMP^58^ for visualization and to search in the PLACE^35^ and JASPAR^34^ databases for potential matches.

Motif cluster averages were mapped to the promoters of differentially expressed genes using the ‘Sitemap’ tool provided in TAMO. The same approach was used to map the previously reported NRE motif. Mapping results were visualized using the ‘GenomeDiagram’ tool included in BioPython v.1.61^59^. Diagrams for visualization of nucleotide frequencies in motifs were all created using Weblogo v.2.8^60^.

To facilitate the comparison of the promoters of genes with similar biological function, differentially expressed genes were annotated using GO terms based on the results obtained by Amar *et al.*^28^. Additional KEGG pathway information for differentially expressed genes, when available, was retrieved from the ‘SolCyc’ database for *S. tuberosum* in the Sol Genomics Network^12^.

## Additional Information

**How to cite this article**: Gálvez, J. H. *et al.* The nitrogen responsive transcriptome in potato (*Solanum tuberosum* L.) reveals significant gene regulatory motifs. *Sci. Rep.* **6**, 26090; doi: 10.1038/srep26090 (2016).

## Supplementary Material

Supplementary Tables

## Figures and Tables

**Table 1:** Experimental design for sampling the potato experiment at the Fredericton Research and Development Centre of Agriculture and Agri-Food Canada, Fredericton NB.

*S. tuberosum* cultivar	Control (N-deficient)[0 kg N ha^−1^]		Treatment (N sufficient)[180 kg N ha^−1^]	
	Time-point 1*July 25, 2012*	Time-point 2*Aug. 8, 2012*	Time-point 1*July 25, 2012*	Time-point 2*Aug. 8, 2012*
Shepody	R1, R2, R3, R4	R1, R2, R3, R4	R1, R2, R3, R4	R1, R2, R3, R4
Russet Burbank	R1, R2, R3, R4	R1, R2, R3, R4	R1, R2, R3, R4	R1, R2, R3, R4
Atlantic	R1, R2, R3, R4	R1, R2, R3, R4	R1, R2, R3, R4	R1, R2, R3, R4

R1, R2, R3 and R4: Biological replicates each consisting of a pool of 15 randomly selected plants from each plot collected at 0800 h, 1100 h, 1400 h and 1700 h, respectively.

**Table 2:** Two-factor Analysis of Variance for phenotypic changes in potato grown under different N supplementation treatments.

		SPAD reading ^a^	Petiole nitrate concentration^a^ [mg g^−1^]	Plant dry matter accumulation ^b^[t ha^−1^]	Fresh tuber yield^b^[t ha^−1^]
N treatment [kg N ha^−1^]					
0		34.34	2.01	8.37	35.32
180		36.85	23.1	9.61	39.29
Cultivar					
Russet Burbank		37.81	13.28	8.37	36.36
Shepody		33.83	12.95	8.66	33.6
Atlantic		35.16	11.43	9.95	41.96
Statistical Significance^$^					
N treatment [N]	*df* = 1	<0.0001***	<0.0001***	0.009**	0.016*
Cultivar [C]	*df* = 2	<0.0001***	0.066	0.016*	<0.001***
N × C	*df* = 2	0.01*	0.92	0.99	0.85

^a^Average of measurements made at two sampling dates (n = 48).

^b^Average of measurements made at harvest (n = 24).

^$^Two-factor ANOVA. Significance codes: ***<0.001 **<0.01 *<0.05.

**Table 3:** Genes found to be consistently over-expressed in plants grown with supplemental N across three cultivars and two sampling dates.

GeneID and Coordinates		Description and InterPro Domains$	GO Terms°	E.C. Numbers and KEGG Pathways
Sotub12g027600 **chr12:64270939–64272220**	−	Whole genome shotgun assembly reference scaffold set scaffold scaffold_4 IPR012336 Thioredoxin-like fold	**BP: CC:** GO:0044444, GO:0043231 **MF:**	
Sotub02g033060 **chr02:66207353–66223198**	+	NAD-dependent epimerase/dehydratase IPR016040 NAD(P)-binding domain	**BP:** GO:0044237 **CC:** GO:0016021,GO:0044444, GO:0043231 **MF:** GO:0050662,GO:0003824	
Sotub10g014450 **chr10:26464815–26466682**	−	Phenylcoumaran benzylic ether reductase 3 IPR008030 NmrA-like	**BP:** GO:0055114, GO:0046686, GO:0044237,GO:0006694 **CC:** GO:0005737 **MF:**GO:0000166, GO:0050662, GO:0003854	**E.C.:** 1.3.1.45
Sotub04g026530 **chr04:53765574–53768766**	−	Peroxidase IPR002016 Haem peroxidase, plant/fungal/bacterial	**BP:** GO:0055114, GO:0042744 **CC:**GO:0009506, GO:0009505, GO:0005773,GO:0005576 **MF:** GO:0004601, GO:0020037	**E.C.:** 1.11.1.7
Sotub08g024220 **chr08:39427772–39430543**	+	Inositol 2-dehydrogenase like protein IPR016040 NAD(P)-binding domain	**BP:** GO:0055114 **CC:** GO:0005576 **MF:**GO:0050112	
Sotub08g007240 **chr08:2517348–2519038**	+	Cation transport regulator-like protein 2 IPR006840 ChaC-like protein	**BP:** GO:0046686, GO:0010288 **CC: MF:**	
Sotub12g011100 **chr12:5784211–5789157**	+	Aminotransferase-like protein IPR005814 Aminotransferase class-III	**BP:** GO:0010154, GO:0046686, GO:0006979,GO:0010183, GO:0010033, GO:0009865,GO:0009450, GO:0006540, GO:0019484 **CC:**GO:0005886, GO:0005739, GO:0009507 **MF:**GO:0030170, GO:0008270, GO:0050897,GO:0003992, GO:0034387	
Sotub10g018540 **chr10:43448319–43452146**	+	Aminotransferase like protein IPR005814 Aminotransferase class-III	**BP:** GO:0050896, GO:0009853 **CC:**GO:0005739 **MF:** GO:0030170, GO:0008453	**E.C.:** 2.6.1.18 **Pathways:** pantothenate and coenzyme A biosynthesis II, β-alanine biosynthesis II **E.C.:** 2.6.1.44 **Pathways:** glycine biosynthesis III
Sotub08g014020 **chr08:22405555–22412221**	+	Chalcone isomerase IPR016087 Chalcone isomerase	**BP: CC:** GO:0009570 **MF:** GO:0016872,GO:0005504	
Sotub01g005580 **chr01:427688–431987**	−	Glutamate decarboxylase IPR010107 Glutamate decarboxylase	**BP:** GO:0006536, GO:0046686 **CC:**GO:0005634, GO:0005829 **MF:** GO:0030170,GO:0004351, GO:0005516	**E.C.:** 4.1.1.15 **Pathways:** glutamate degradation IV, glutamate degradation IX (via 4-aminobutyrate), glutamate dependent acid resistance
Sotub10g024560 **chr10:49073980–49076488**	+	Glutathione S-transferase IPR004046 Glutathione S-transferase, C-terminal	**BP:** GO:0046686, GO:0009636 **CC:**GO:0005829 **MF:** GO:0005515, GO:0004364	**E.C.:** 2.5.1.18 **Pathways:** glutathione-mediated detoxification II
Sotub06g008080 **chr06:4763763–4768618**	−	Male sterility 5 family protein (Fragment) IPR011990 Tetratricopeptide-like helical	**BP: CC: MF:** GO:0005515	
Sotub09g018850 **chr09:37905080–37908059**	+	Male sterility 5 family protein (Fragment) IPR011990 Tetratricopeptide-like helical	**BP: CC: MF:** GO:0005515	
Sotub01g022620 **chr01:69840666–69841253**	−	Peptide methionine sulfoxide reductase msrB IPR002579 Methionine sulphoxide reductase B	**BP:** GO:0022900 **CC:** GO:0005829 **MF:**GO:0046872, GO:0033743, GO:0008113	**E.C.:** 1.8.4.11
Sotub05g024960 **chr05:56803278–56805488**	−	Amino acid transporter IPR013057 Amino acid transporter, transmembrane	**BP:** GO:0006865 **CC:** GO:0016021 **MF:**GO:0003674	
Sotub11g012150 **chr11:6925093–6928544**	−	Amino acid transporter IPR013057 Amino acid transporter, transmembrane	**BP: CC:** GO:0005886, GO:0016021,GO:0005774 **MF:** GO:0015171	
Sotub02g036900 **chr02:69051466–69053337**	+	Cystine transporter Cystinosin IPR005282 Lysosomal cystine transporter	**BP:** GO:0006810 **CC:** GO:0005886, GO:0016021, GO:0005765 **MF:**	
Sotub09g024290 **chr09:46805115–46809993**	−	Sulfate adenylyltransferase IPR002650 ATP-sulfurylase	**BP:** GO:0046686, GO:0001887, GO:0000103,GO:0070814 **CC:** GO:0005886, GO:0005739,GO:0009570 **MF:** GO:000552, GO:0004781	**E.C.:** 2.7.7.4 **Pathways:** selenate reduction, sulfate reduction II (assimilatory), sulfate activation for sulfonation
Sotub04g021910 **chr04:45794993–45803770**	+	Sulfate transporter IPR001902 Sulphate anion transporter	**BP:** GO:0055085, GO:0030003, GO:0019344,GO:0009684, GO:0019761, GO:0070838,GO:0008272 **CC:** GO:0016021 **MF:**GO:0015293, GO:0008271	
Sotub04g027100 **chr04:54585161–54588369**	−	High affinity sulfate transporter 2 PR001902 Sulphate anion transporter	**BP:** GO:0055085, GO:0009970, GO:0008272,GO:0080160 **CC:** GO:0005886, GO:0016021**MF:** GO:0015293, GO:0008271	
Sotub10g013960 **chr10:24455891–24459003**	−	High affinity sulfate transporter 2 IPR001902 Sulphate anion transporter	**BP:** GO:0055085, GO:0009970, GO:0008272,GO:0080160 **CC:** GO:0005886, GO:0016021**MF:** GO:0008271	
Sotub08g005390 **chr08:414965–419119**	+	Nitrate transporter IPR000109 TGF-beta receptor, type I/II extracellular region	**BP:** GO:0009414, GO:0010167, GO:0009734,GO:0009635, GO:0006857, GO:0042128 **CC:**GO:0005886, GO:0016021 **MF:** GO:0015112,GO:0015293	
Sotub07g009860 **chr07:6361828–6364633**	+	Peptide transporter IPR000109 TGF-beta receptor, type I/II extracellular region	**BP:** GO:0009987, GO:0015031, GO:0006807,GO:0006857 **CC:** GO:0016021, GO:0009506**MF:** GO:0042937, GO:0042936	
Sotub01g049270 **chr01:96560230–96568031**	−	Tyrosine-protein kinase transforming protein Src IPR015783 ATMRK serine/threonine protein kinase-like	**BP:** GO:0006468 **CC:** GO:0016597,GO:0005524, GO:0004674, GO:0004715 **MF:**	
Sotub03g018720 **chr03:24381058–24384772**	+	Alpha-glucosidase-like IPR000322 Glycoside hydrolase, family 31	**BP:** GO:0005975 **CC:** GO:0030246,GO:0032450 **MF:**	
Sotub01g023000 **chr01:70600749–70601999**	+	Xylanase inhibitor (Fragment) IPR001461 Peptidase A1	**BP:** GO:0006508 **CC:** GO:0004190 **MF:**	
Sotub11g007110 **chr11:2283766–2284773**	+	Plant-specific domain TIGR01615 family protein IPR006502 Protein of unknown function DUF506, plant	**BP: CC: MF:**	
Sotub11g007090 **chr11:2266700–2267713**	+	Plant-specific domain TIGR01615 family protein IPR006502 Protein of unknown function DUF506, plant	**BP: CC: MF:**	
Sotub04g023170 **chr04:484 12542–48413816**	+	Unknown Protein	**BP: CC: MF:**	
Sotub03g017290 **chr03:22560550–22560888**	−	Unknown Protein	**BP: CC: MF:**	

^$^Gene descriptions (including InterPro domains) obtained from the ITAG1.0 annotation system^11^. Strand: plus (+) or minus (−).

^°^BP: Biological Process; CC: Cell Component; MF: Molecular function.

**Table 4:** Genes found to be consistently under-expressed in plants grown with supplemental N across three cultivars and two sampling dates.

GeneID and Coordinates		Description$	GO Terms°	E.C. Numbers and KEGG Pathways
Sotub12g012740** **chr12:7720521–7725325**	+	Chloroplast lipocalin IPR000566 Lipocalin-related protein and Bos/Can/Equ allergen	**BP:** GO:0006979 **CC:** GO:0009535, GO:0005576,GO:0031977 **MF:** GO:0005488	
Sotub02g033320 **chr02:66437615–66439427**	+	Proline dehydrogenase IPR015659 Proline oxidase	**BP:** GO:0055114, GO:0006979, GO:0009414,GO:0006970, GO:0006537, GO:0010133 **CC:**GO:0005739 **MF:** GO:0004657	**E.C.:** 1.5.1.2 **Pathways:** proline biosynthesis I, II (from argininte), and III, arginine degradation VI (arginase 2 pathway) **E.C.:** 1.5.99.8 **Pathways:** L-Nδ-acetylornithine biosynthesis, proline degradation
Sotub08g025870* **chr08:40745121–40749856**	+	Primary amine oxidase IPR000269 Copper amine oxidase	**BP:** GO:0055114, GO:0009738, GO:0009308,GO:0006809 **CC:** GO:0005768, GO:0005802,GO:0005773 **MF:** GO:0005507, GO:0048038,GO:0008131, GO:0052596, GO:0052595,GO:0052594, GO:0052593	**E.C.:** 1.4.3.22, 1.4.3.21 **Pathways**: phenylethanol biosynthesis
Sotub09g009440 **chr09:6333813–6338909**	+	Cation/H+ antiporter IPR006153 Cation/H+ exchanger	**BP:** GO:0055085, GO:0006623, GO:0006813,GO:0006885, GO:0030007, GO:0030104 **CC:**GO:0016021, GO:0005783, GO:0009507,GO:0012505 **MF:** GO:0015385	
Sotub09g023510 **chr09:45964253–45969159**	+	High affinity sulfate transporter 2 IPR001902 Sulphate anion transporter	**BP:** GO:0055085, GO:0006950, GO:0008272 **CC:**GO:0016021, GO:0009507 **MF:** GO:0015293,GO:0008271	
Sotub02g017430* **chr02:51710764–51712899**	−	Purine permease family protein IPR004853 Protein of unknown function DUF250	**BP:** GO:0016021 **CC: MF:**	
Sotub05g028860 **chr05:60345721–60347082**	−	Flowering locus T protein IPR008914 Phosphatidylethanolamine-binding protein PEBP	**BP:** GO:0009909, GO:0048510, GO:0010229,GO:0030154, GO:0048575 **CC:** GO:0005737,GO:0005634 **MF:** GO:0008429	
Sotub12g031130 **chr12:67026510–67029847**	+	Poly(A) polymerase IPR007012 Poly(A) polymerase, central region	**BP:** GO:0006351, GO:0043631 **CC:** GO:0005737,GO:0005634 **MF:** GO:0003723, GO:0004652	
Sotub01g049920 **chr01:97188627–97192358**	−	Nodule inception protein (Fragment) IPR003035 Plant regulator RWP-RK	**BP:** GO:0006355 **CC:** GO:0005634 **MF:** GO:0003677	
Sotub12g020880 **chr12:54796545–54800381**	−	Ubiquinone/menaquinone biosynthesis methyltransferase ubiE IPR013216 Methyltransferase type 11	**BP:** GO:0032259, GO:0009860, GO:0009877,GO:0009555, GO:0048528, GO:0010183,GO:0009312, GO:0006656, GO:0042425 **CC:**GO:0005829 **MF:** GO:0000234	**E.C.:** 2.1.1.103 P**athways:** superpathway of choline biosynthesis, phosphatidylcholine biosynthesis II, choline biosynthesis I
Sotub05g012720 **chr05:7174173–7177870**	−	Nodulin MtN21 family protein IPR000620 Protein of unknown function DUF6, transmembrane	**BP: CC:** GO:0016020 **MF:**	
Sotub09g029950 **chr09:52340673–52341549**	+	Cell wall protein IPR010800 Glycine rich	**BP: CC: MF:**	
Sotub09g010630* **chr09:7495968–7498403**	+	Hydrolase alpha/beta fold family protein IPR000073 Alpha/beta hydrolase fold-1	**BP: CC: MF:**	

^$^Gene descriptions (including InterPro domains) obtained from the ITAG1.0 annotation system^11^. Strand: plus (+) or minus (−).

^°^BP: Biological Process; CC: Cell Component; MF: Molecular function.

^*^Genes found to be significantly under-expressed only in the first sampling date (2012-07-25).

^**^Genes found to be significantly under-expressed only in the second sampling date (2012-08-08).

**Table 5:**
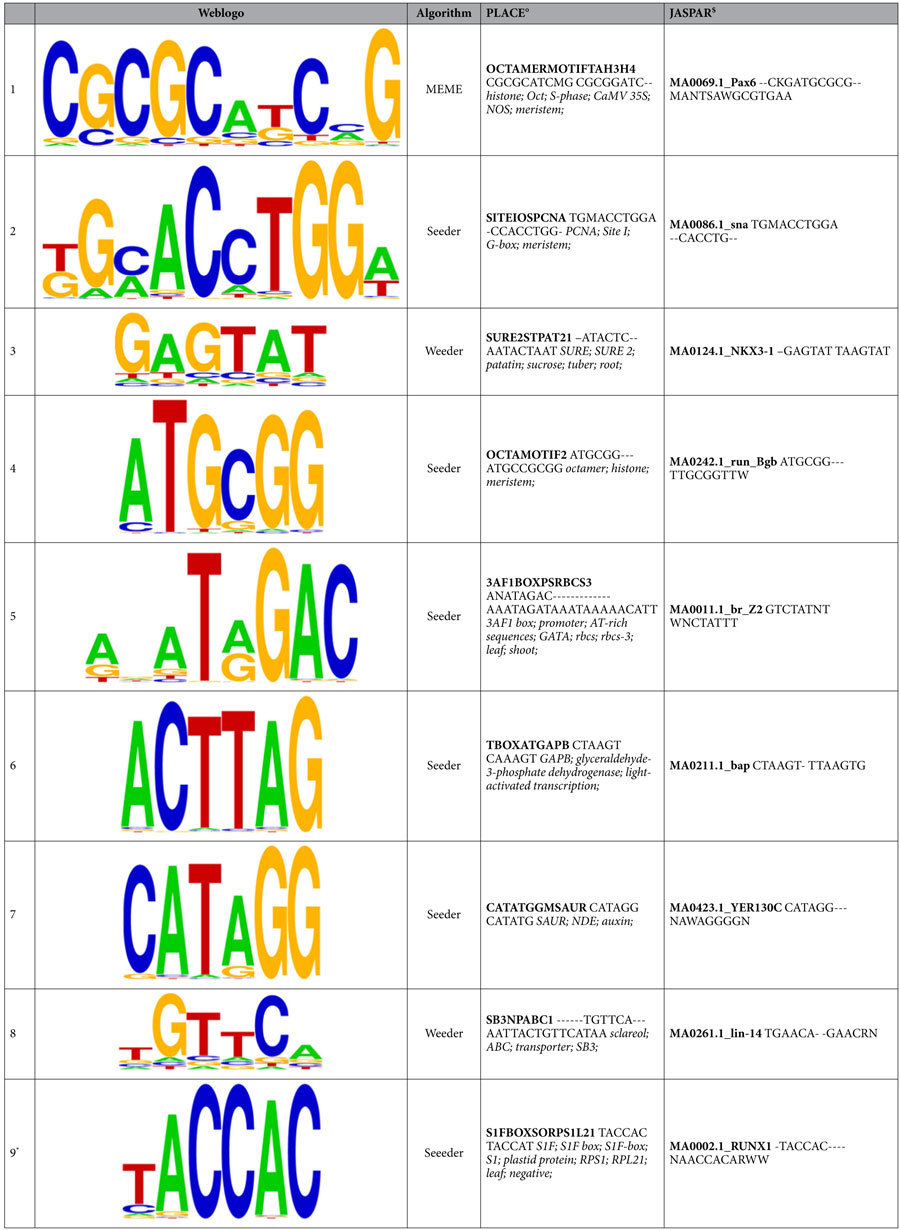
Nine regulatory motifs discovered in the upstream flanking region of N responsive genes.

^*^Motif discovered in the upstream regions of under-expressed genes.

^°^Best match in PLACE database^35^: motif accession code in bold; alignment (top: predicted motif, bottom: motif found in database); keywords associated with the motif (in italics).

^$^Best match in JASPAR database^34^: motif accession code in bold; alignment (top: predicted motif, bottom: motif found in database).
