# Supplementary material for: The nitrogen responsive transcriptome in potato (Solanum tuberosum L.) reveals significant gene regulatory motifs
Source: Sci Rep. 2016 May 19;6:26090. doi: 10.1038/srep26090 (PMC4872257; doi:10.1038/srep26090)
Supplement: Supplementary Tables [file srep26090-s1.pdf]

The nitrogen responsive transcriptome in potato (*Solanum tuberosum* L.) reveals significant gene regulatory motifs

José Héctor Gálvez, Helen H. Tai, Martin Lague, Bernie J. Zebarth, and Martina V. Strömvik

Supplementary Table 1: Petiole nitrate concentrations and SPAD measurements for all cultivars and both time-points.

Date	Cultivar	N rate [kg N ha^{-1}]	Replicate Number	Petiole Nitrate Concentration [mg/g]	SPAD readings
2012-07-25	Russet Burbank	0	R1	1.2	35.8
2012-07-25	Russet Burbank	0	R2	3.4	38.7
2012-07-25	Russet Burbank	0	R3	3.0	37.1
2012-07-25	Russet Burbank	0	R4	8.8	39.5
2012-07-25	Russet Burbank	180	R1	25.5	37.4
2012-07-25	Russet Burbank	180	R2	25.1	37.3
2012-07-25	Russet Burbank	180	R3	23.8	37.2
2012-07-25	Russet Burbank	180	R4	27.4	40.4
2012-07-25	Shepody	0	R1	3.3	36.0
2012-07-25	Shepody	0	R2	4.1	32.5
2012-07-25	Shepody	0	R3	0.7	30.7
2012-07-25	Shepody	0	R4	4.6	34.2
2012-07-25	Shepody	180	R1	24.4	33.3
2012-07-25	Shepody	180	R2	25.4	37.8
2012-07-25	Shepody	180	R3	22.9	38.5
2012-07-25	Shepody	180	R4	23.3	38.6
2012-07-25	Atlantic	0	R1	0.5	33.5
2012-07-25	Atlantic	0	R2	1.9	37.0
2012-07-25	Atlantic	0	R3	1.0	35.7
2012-07-25	Atlantic	0	R4	2.0	37.4
2012-07-25	Atlantic	180	R1	22.2	35.7
2012-07-25	Atlantic	180	R2	18.0	35.1
2012-07-25	Atlantic	180	R3	21.5	36.4
2012-07-25	Atlantic	180	R4	18.8	36.8
2012-08-08	Russet Burbank	0	R1	0.3	34.9
2012-08-08	Russet Burbank	0	R2	0.4	37.2
2012-08-08	Russet Burbank	0	R3	0.9	36.4
2012-08-08	Russet Burbank	0	R4	5.3	38.6
2012-08-08	Russet Burbank	180	R1	23.1	37.0
2012-08-08	Russet Burbank	180	R2	23.5	38.8
2012-08-08	Russet Burbank	180	R3	21.6	38.8
2012-08-08	Russet Burbank	180	R4	19.4	39.8
2012-08-08	Shepody	0	R1	2.4	30.3
2012-08-08	Shepody	0	R2	1.8	31.8
2012-08-08	Shepody	0	R3	0.4	26.2
2012-08-08	Shepody	0	R4	0.8	28.8
2012-08-08	Shepody	180	R1	22.8	35.0
2012-08-08	Shepody	180	R2	23.2	35.6
2012-08-08	Shepody	180	R3	23.3	36.8
2012-08-08	Shepody	180	R4	23.9	35.1
2012-08-08	Atlantic	0	R1	0.2	31.5
2012-08-08	Atlantic	0	R2	0.2	33.6
2012-08-08	Atlantic	0	R3	0.2	33.9
2012-08-08	Atlantic	0	R4	0.7	32.8
2012-08-08	Atlantic	180	R1	21.4	36.0
2012-08-08	Atlantic	180	R2	26.5	34.9
2012-08-08	Atlantic	180	R3	28.5	35.7
2012-08-08	Atlantic	180	R4	19.5	36.5

Supplementary Table 2: Plant dry biomass and fresh tuber yields for all cultivars at harvest.

**Cultivar**	**N rate** [kg N ha^{-1}]	**Replicate Number**	**Plant Dry Biomass** [t/ha]	**Fresh Tuber Yield** [t/ha]
Russet Burbank	0	R1	6.82	36.0
Russet Burbank	0	R2	8.64	33.7
Russet Burbank	0	R3	6.98	37.6
Russet Burbank	0	R4	8.45	29.8
Russet Burbank	180	R1	7.75	34.8
Russet Burbank	180	R2	8.03	44.9
Russet Burbank	180	R3	9.74	31.1
Russet Burbank	180	R4	10.54	42.9
Shepody	0	R1	8.81	32.6
Shepody	0	R2	7.86	28.3
Shepody	0	R3	7.18	28.1
Shepody	0	R4	8.42	35.5
Shepody	180	R1	8.29	35.6
Shepody	180	R2	10.03	37.3
Shepody	180	R3	9.90	36.9
Shepody	180	R4	8.74	34.4
Atlantic	0	R1	7.81	37.0
Atlantic	0	R2	11.11	43.1
Atlantic	0	R3	9.81	39.8
Atlantic	0	R4	8.53	42.4
Atlantic	180	R1	9.95	45.7
Atlantic	180	R2	11.42	43.2
Atlantic	180	R3	10.64	41.1
Atlantic	180	R4	10.31	43.4

Supplementary Table 3: Total number of differentially expressed genes for different potato cultivars.

***S. tuberosum* cultivar**	**Time-point 1 July 25, 2012**		**Time-point 2 Aug. 8, 2012**	
	Over-expressed	Under-expressed	Over-expressed	Under-expressed
**Shepody**	182	35	218	52
**Russet Burbank**	64	47	116	18
**Atlantic**	393	33	149	40

Supplementary Table 4: Genes that were differentially expressed in only one of the two time points.

Time point	Gene ID	Gene Description and Interpro Domain$
**Over expressed genes**		
2012-07-25	Sotub01g007180	AMP-dependent synthetase and ligase; IPR011614 Catalase, N-terminal
2012-07-25	Sotub02g012390	Coiled-coil domain-containing protein 109A; IPR006769 Protein of unknown function DUF607
2012-07-25	Sotub03g018730	Glutamate dehydrogenase; IPR014362 Glutamate dehydrogenase
2012-07-25	Sotub03g023340	BTB/POZ domain-containing protein; IPR000197 Zinc finger, TAZ-type
2012-07-25	Sotub03g031100	Heat shock protein; IPR013126 Heat shock protein 70
2012-07-25	Sotub04g025700	ASR4 protein (Fragment); IPR003496 ABA/WDS induced protein
2012-07-25	Sotub04g035440	Cellular retinaldehyde-binding/triple function C-terminal; IPR001251 Cellular retinaldehyde-binding/triple function, C-terminal
2012-07-25	Sotub05g007580	Myb family transcription factor; IPR006447 Myb-like DNA-binding region, SHAQKYF class
2012-07-25	Sotub05g008150	3-ketoacyl-CoA synthase; IPR012392 Very-long-chain 3-ketoacyl-CoA synthase
2012-07-25	Sotub06g006790	Plant-specific domain TIGR01615 family protein; IPR006502 Protein of unknown function DUF506, plant
2012-07-25	Sotub06g011740	Gamma-glutamyl phosphate reductase; IPR005766 Delta l-pyrroline-5-carboxylate synthetase
2012-07-25	Sotub06g023090	Solute carrier family 2, facilitated glucose transporter member 3; IPR003663 Sugar/inositol transporter
2012-07-25	Sotub06g027390	Cysteine proteinase inhibitor; IPR006043 Xanthine/uracil/vitamin C permease
2012-07-25	Sotub08g005550	PII uridylyl-transferase; IPR002912 Amino acid-binding ACT
2012-07-25	Sotub11g007070	Plant-specific domain TIGR01615 family protein; IPR006502 Protein of unknown function DUF506, plant
2012-07-25	Sotub11g007100	Plant-specific domain TIGR01615 family protein; IPR006502 Protein of unknown function DUF506, plant
2012-07-25	Sotub11g020550	Hexokinase 6; IPR001312 Hexokinase
2012-08-08	Sotub02g015720	FAD binding domain-containing protein; IPR006094 FAD linked oxidase, N-terminal
2012-08-08	Sotub02g021000	Plant-specific domain TIGR01615 family protein; IPR006502 Protein of unknown function DUF506, plant
2012-08-08	Sotub02g031260	Arabinogalactan
2012-08-08	Sotub03g012290	Kunitz-type protease inhibitor; IPR002160 Proteinase inhibitor I3, Kunitz legume
2012-08-08	Sotub03g012340	Kunitz-type protease inhibitor; IPR002160 Proteinase inhibitor I3, Kunitz legume
2012-08-08	Sotub03g012360	Proteinase inhibitor II; IPR003465 Proteinase inhibitor I20, Pin2
2012-08-08	Sotub03g015880	Kunitz trypsin inhibitor 4; IPR011065 Kunitz inhibitor ST1-like
2012-08-08	Sotub03g015970	Aspartic protease inhibitor 1; PR002160 Proteinase inhibitor I3, Kunitz legume
2012-08-08	Sotub03g023330	Peptide methionine sulfoxide reductase MsrA; IPR002569 Methionine sulphoxide reductase A
2012-08-08	Sotub03g035920	Taurine catabolism dioxygenase TauD/TfdA; IPR003819 Taurine catabolism dioxygenase TauD/TfdA
2012-08-08	Sotub04g028270	Phospho-2-dehydro-3-deoxyheptonate aldolase 1; IPR002480 DAHP synthetase, class II
2012-08-08	Sotub05g018510	GDSL esterase/lipase At2g04570; IPR001087 Lipase, GDSL
2012-08-08	Sotub05g021450	Glucose-6-phosphate/phosphate translocator 2; IPR004696 Tpt phosphate/phosphoenolpyruvate translocator
2012-08-08	Sotub05g027780	High affinity sulfate transporter 2; IPR001902 Sulphate anion transporter
2012-08-08	Sotub06g025010	Cortical cell-delineating protein; IPR013770 Plant lipid transfer protein and hydrophobic protein, helical
2012-08-08	Sotub06g026740	Chlorophyll a-b binding protein 4, chloroplastic; IPR001344 Chlorophyll A-B binding protein
2012-08-08	Sotub06g027990	Unknown Protein
2012-08-08	Sotub06g030410	Beta-D-glucosidase; IPR001764 Glycoside hydrolase, family 3, N-terminal
2012-08-08	Sotub06g030610	Cysteine proteinase inhibitor; IPR000010 Proteinase inhibitor I25, cystatin
2012-08-08	Sotub07g016530	Cellulose synthase-like protein H1; IPR005150 Cellulose synthase
2012-08-08	Sotub07g016550	UDP glucosyltransferase; IPR002213 UDP-glucuronosyl/UDP-glucosyltransferase
2012-08-08	Sotub07g016570	1-aminocyclopropane-1-carboxylate oxidase; IPR005123 Oxoglutarate and iron-dependent oxygenase
2012-08-08	Sotub08g024210	Exostosin family protein; IPR004263 Exostosin-like

2012-08-08	Sotub08g028270	Methanol inducible protein
2012-08-08	Sotub09g008430	Threonine dehydratase biosynthetic; IPR005787 Threonine dehydratase I
2012-08-08	Sotub09g023600	Homocysteine s-methyltransferase; IPR003726 Homocysteine S-methyltransferase
2012-08-08	Sotub09g026640	Proteinase inhibitor I; IPR000864 Proteinase inhibitor I13, potato inhibitor I
2012-08-08	Sotub09g028690	Selenium binding protein; IPR008826 Selenium-binding protein
2012-08-08	Sotub09g031120	Unknown Protein; IPR006706 Extensin-like region
2012-08-08	Sotub10g021050	UDP glucosyltransferase; IPR002213 UDP-glucuronosyl/UDP-glucosyltransferase
2012-08-08	Sotub11g024220	Superoxide dismutase; IPR001424 Superoxide dismutase, copper/zinc binding
2012-08-08	Sotub12g007850	Cytosol aminopeptidase family protein; IPR011356 Peptidase M17, leucyl aminopeptidase
2012-08-08	Sotub12g008260	Unknown Protein
2012-08-08	Sotub12g028670	Cation transport regulator-like protein 2; IPR006840 ChaC-like protein
**Under expressed genes**		
2012-07-25	Sotub02g017430	Purine permease family protein; IPR004853 Protein of unknown function DUF250
2012-07-25	Sotub08g025870	Primary amine oxidase; IPR000269 Copper amine oxidase
2012-07-25	Sotub09g010630	Hydrolase alpha/beta fold family protein; IPR000073 Alpha/beta hydrolase fold-1
2012-08-08	Sotub12g012740	Chloroplast lipocalin; IPR000566 Lipocalin-related protein and Bos/Can/Equ allergen

$ Gene descriptions (including InterPro domains) obtained from the ITAG1.0 annotation system (The Tomato Genome Consortium 2012)
